# Understanding the ontogeny of foraging behaviour: insights from combining marine predator bio-logging with satellite-derived oceanography in hidden Markov models

**DOI:** 10.1098/rsif.2018.0084

**Published:** 2018-06-06

**Authors:** W. James Grecian, Jude V. Lane, Théo Michelot, Helen M. Wade, Keith C. Hamer

**Affiliations:** 1 School of Biology, Faculty of Biological Sciences, University of Leeds, Leeds LS2 9JT, UK; 2 Sea Mammal Research Unit, Scottish Oceans Institute, University of St Andrews, St Andrews KY16 8LB, UK; 3 School of Mathematics and Statistics, University of Sheffield, Sheffield S3 7RH, UK; 4 Scottish Natural Heritage, Battleby, Redgorton, Perth PH1 3EW, UK

**Keywords:** animal telemetry, foraging ecology, finite-size Lyapunov exponent, learning, marine vertebrate, movement ecology

## Abstract

The development of foraging strategies that enable juveniles to efficiently identify and exploit predictable habitat features is critical for survival and long-term fitness. In the marine environment, meso- and sub-mesoscale features such as oceanographic fronts offer a visible cue to enhanced foraging conditions, but how individuals learn to identify these features is a mystery. In this study, we investigate age-related differences in the fine-scale foraging behaviour of adult (aged ≥ 5 years) and immature (aged 2–4 years) northern gannets *Morus bassanus*. Using high-resolution GPS-loggers, we reveal that adults have a much narrower foraging distribution than immature birds and much higher individual foraging site fidelity. By conditioning the transition probabilities of a hidden Markov model on satellite-derived measures of frontal activity, we then demonstrate that adults show a stronger response to frontal activity than immature birds, and are more likely to commence foraging behaviour as frontal intensity increases. Together, these results indicate that adult gannets are more proficient foragers than immatures, supporting the hypothesis that foraging specializations are learned during individual exploratory behaviour in early life. Such memory-based individual foraging strategies may also explain the extended period of immaturity observed in gannets and many other long-lived species.

## 1. Introduction

The mortality of young animals is typically much higher than that of adults and explaining this difference is fundamental to the study of population age structure, dynamics and persistence [1,2]. The main hypothesis invoked to explain higher mortality among immatures is a lack of proficiency in skills such as foraging and predator avoidance, due to a lack of experience and learning combined with physical immaturity [3–5]. Inequalities in levels of foraging ability may result in young animals being competitively excluded from optimal foraging habitat by more experienced adults [5,6]. Alternatively, young animals may lack the experience to recognize profitable patches [7]. This could lead to the selective disappearance of immatures incapable of developing appropriate foraging skills [8,9] and may explain why many long-lived iteroparous animals delay the age of first breeding until well after they become physiologically mature [10–12].

Individual foraging specializations are prevalent among adults of long-lived species [13,14], and have potentially far-reaching consequences for individual fitness, as well as influencing the manner in which populations can respond to environmental change [15]. However, the mechanisms producing and maintaining such individual differences are only poorly understood. In some species, foraging specializations are learned by cultural transmission from mother to offspring (e.g. in sea otters *Enhydra lutris* [16]) or among a close-knit social group (e.g. in social primates and dolphins [17,18]). However, in most cases individuals acquire foraging specializations independently and in the absence of detectable morphological differences. Hence an alternative explanation is that such specializations are learned during individual exploratory behaviours in early life, that then become canalized and refined with age and experience [14,19]. This ‘exploration-refinement’ process [20] may be especially important for some forms of specialization such as individual foraging site fidelity (IFSF), where an animal repeatedly visits the same foraging patch. However, there are very few data to examine the development of IFSF [19] or the association between IFSF and foraging proficiency. IFSF could result from individuals learning to identify and relocate profitable patches, but while it is well known that foraging competence tends to increase with age [5,10] it is less clear whether or not this includes an enhanced ability to recognize suitable patches.

In the marine environment, meso- and sub-mesoscale oceanic features such as fronts, eddies and filaments entrain nutrients, enhance primary productivity and aggregate zooplankton [21–23]. These features occur throughout the oceans, creating enhanced foraging conditions that attract higher predators, including cetaceans [24], sea turtles [25], pinnipeds [26] and seabirds [27]. The foraging behaviour of these marine predators has been linked to fronts identified from both composite mapping of remotely sensed sea surface temperature and chlorophyll-*a* fields [28–30], and from surface velocity fields estimated via satellite altimetry [27,31]. However, while these features are ubiquitous, spatial and temporal variation in size, intensity and persistence affects their suitability as foraging patches [28,32], and we lack an understanding of how individuals learn to identify these areas or the cues that they use to find them.

In this study, our objective was to investigate simultaneous age-related differences in both foraging specialization (IFSF) and proficiency; in particular, the use of frontal areas as foraging habitat. We focus on the northern gannet *Morus bassanus* (hereafter gannet), a long-lived neritic seabird characterized by over-lapping generations and a long pre-breeding period (≥5 years) [33]. Adult gannets display high consistency in individual foraging behaviour [34,35] including IFSF associated with foraging in areas of high frontal activity [28,29]. By contrast, a recent study revealed much lower levels of IFSF among immature birds, suggesting that young individuals require a protracted period of learning to develop the foraging consistency observed in adults [19]. Alternatively, immature birds may simply choose to explore a greater range of different sites than adults on successive foraging trips. It is not currently known whether immatures are less able than adults to locate and exploit areas of high frontal activity or whether IFSF is associated with a more restricted foraging distribution among adults overall, as might be expected if birds learn to avoid unprofitable foraging areas.

To better understand how cognitive processes are influenced by and give rise to movement patterns requires the integration of high-resolution telemetry data, fine-scale remote sensing data and recent methodological developments in data analysis [36]. Here, we combine data collected by high-resolution GPS-loggers with satellite-derived measures of frontal activity using state-switching models [37]. We compare the foraging specialization and proficiency of immature and chick-rearing adult gannets, examining three specific predictions: (i) adults use a more restricted range of foraging locations than immature birds, resulting in a narrower foraging distribution at population level and hence a degree of segregation between adults and immature birds at sea; (ii) adults show both higher IFSF and a stronger response than immature birds to areas of high frontal activity indicative of suitable foraging sites and (iii) associated with these changes, adults make more effective use of time at sea, spending no more time foraging than immatures, despite needing to provide for dependent offspring in addition to themselves.

## 2. Methods

### 2.1. Study system and data collection

Fieldwork was conducted between June and August 2015 at the world's largest gannet colony, Bass Rock, Scotland (56°60 N, 2°36 W), where *ca* 75 000 pairs breed annually. Using a 6 m telescopic pole fitted with a wire crook, 35 adult gannets (ages ≥ 5 years) were caught at the nest-site while attending chicks, and 21 immature gannets (ages 2–4 years, identified using plumage characteristics [33,38]) were caught at club sites (areas of the colony frequented by pre-breeding individuals) or while attempting to hold territories around the colony. On capture, birds were marked with a unique metal ring (British Trust for Ornithology, UK) and an individually numbered colour-ring [39]. We deployed GPS-loggers (i-gotU GT-600, Mobile Action Technology Inc., Taipei, Taiwan, 37 g) on adult birds and GPS Radio Frequency loggers (GPS-RF, e-obs GmbH, Munich, Germany, 45 g) on immature birds as recapture was unlikely but remote download of the data was possible within 2 km of the colony. All loggers were attached to the upper side of three central tail feathers using Tesa^©^ tape, and programmed to record locations every 2 min. Total handling time was approximately 15 min. Maximum device weight (45 g) was less than 2% of body weight (3.2 ± 0.3 kg) and below the maximum recommended for bio-logging studies [40], while the difference in device weights for adults and immature birds was only 0.25% of body mass. Previous studies indicate that such deployments have no discernible impact on trip durations or body masses of birds [41,42]. We recaptured 34 adults, providing 31 devices with usable data, and downloaded usable datasets from 15 immature birds.

### 2.2. Oceanographic data

To identify areas of frontal activity, we used the backward-in-time finite-size Lyapunov exponent (FSLE, [43]) available via CLS/CNES Aviso (http://www.aviso.altimetry.fr). This technique measures the relative dispersion of particles traced over altimetry-derived time-dependent current velocity fields [43]. Ridges of high FSLE values occur where formally distant water masses converge to create a transport front, providing a good proxy for areas of frontal activity such as sub-mesoscale chlorophyll and SST filaments [44]. As a Lagrangian diagnostic, this approach has the benefit of (i) incorporating both the spatial and temporal variability of altimetry velocity fields [24] and (ii) approximating the types of Lagrangian coherent structures that marine predators have previously been shown to exploit [27,45,46].

### 2.3. Statistical analysis

During data processing, we defined foraging trips as periods when birds were more than 10 km from the colony for more than 40 min; all other locations were classified as either colony attendance or rafting [47] and excluded from this analysis. All data were transformed to a UTM 30N projection and, to remove irregularities in satellite uplink time, were regularized by linear interpolation to 2 min intervals using the package adehabitatLT v. 0.3.23 [48].

To quantify the extent to which the foraging distributions of adult and immature birds overlapped, we calculated the bivariate kernel utilization distribution (UD) for each group using a smoothing parameter of 10 km and a grid size of 1 km in the package adehabitatHR v. 0.4.15 [48]. Overlap was estimated using Bhattacharyya's affinity (BA) [49] where 0 equates to no overlap and 1 to complete overlap in the UDs. We estimated a null distribution of BA values by randomly reassigning age class among the 46 individuals 1000 times and calculated *p*-values as the proportion of random assignment BA values that were smaller than the observed BA estimate [42].

For each foraging trip, we calculated: (*a*) trip duration (h), (*b*) total trip length (km), (*c*) departure angle (average of the first five bearings greater than 10 km from the colony, rad), (*d*) trip range (maximum displacement from the colony, km), (*e*) the *x*-coordinate and (*f*) the *y*-coordinate of the furthest location from the colony (m) and (*g*) the trip area (minimum convex polygon, km^2^). Differences in trip characteristics between adult and immature birds were then examined using linear mixed-effects models fitted with bird ID as a random intercept as there were multiple trip measurements per individual. In these models, trip duration, total distance travelled and foraging area were log_10_ transformed.

After testing for population-level differences, we examined the consistency of individual differences in trip characteristics by calculating a measure of repeatability based on the intra-class correlation coefficient from linear mixed-effect models fitted with bird ID as a random intercept using the package rptR [50]. We used repeatability as a proxy for foraging specialization within the adult and immature populations, testing the null hypothesis that between-individual variance in a particular characteristic was equal to within-individual variance [34]. We then tested differences in the repeatability of trip characteristics between adult and immature birds by calculating pairwise differences in *Z*-transformed repeatability estimates (*Zr*) and examined whether or not the corresponding confidence intervals overlapped zero [50,51]. For departure angles, we calculated repeatability using circular ANOVAs fitted with the package circular [52] following standard methods [53,54].

We used hidden Markov models (HMMs) to examine the at-sea behaviour of adult and immature gannets using the package moveHMM v.1.0 [55]. The movement of an individual along a foraging trip was decomposed into three underlying states by characterization of the distributions of step lengths and turning angles between consecutive locations. We used a gamma distribution to describe the step lengths and a von Mises distribution to describe the turning angles. The three states were based on *a priori* understanding of gannet behaviour [56]; during a foraging trip individuals will (i) spend time in directed flight to and from foraging patches, (ii) perform slow and tortuous flight when foraging within a patch, and (iii) spend time resting on the sea surface [57]. During a previous study of gannets equipped with GPS loggers and time–depth recorders (TDRs) 81% of all TDR dives corresponded with locations identified as foraging by a similarly parameterized HMM [57]. As initial parameter values are required for model estimation, we verified that the model had identified the maximum-likelihood estimates of the parameters by refitting the model 25 times with random initial parameter values. We used the Viterbi algorithm to estimate the most likely sequence of movement states to have generated the observations based on the fitted model [58].

To assess differences in movement patterns between adult and immature birds, we included the additive effect of age (binary; adult/immature), FSLE and the interaction between the two as covariates in the HMM framework. These covariates were included within the HMM formulation as a logistic regression that expresses the transition probabilities of the underlying state process as a function of the covariates, allowing us to assess the importance of the covariates on the probability of switching between states [59,60]. FSLE values were transformed to a positive scale to aid interpretation. The resulting models were then ranked based on the Akaike information criterion (AIC). Finally, we examined differences in the proportion of time adult and immature gannets spent in each of the three states using mixed-effects logistic regressions, with bird ID as a random intercept using the package lme4 v. 1.1-10 [61]. All analyses were conducted using R v. 3.2.2 [62].

## 3. Results

### 3.1. Foraging distribution

**Figure 1.**
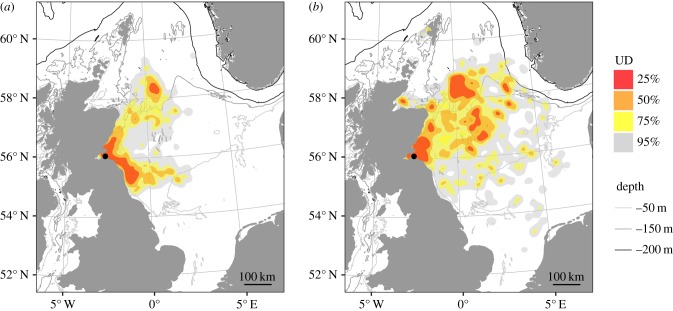
At-sea distribution of (*a*) 31 adult and (*b*) 15 immature gannets estimated from the bivariate kernel utilization distribution (UD) of GPS locations. Colours represent specific UD contours; the breeding colony is represented by a black dot; grey lines represent 50 m, 150 m and 200 m depth contours. (Online version in colour.)

**Figure 2.**
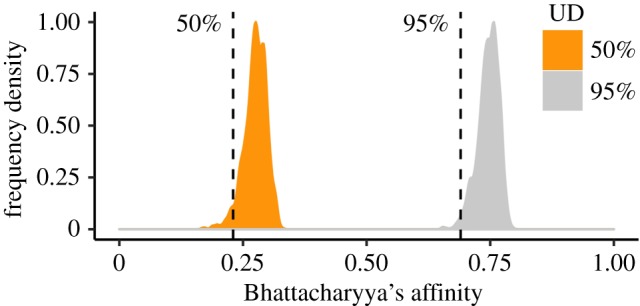
Observed overlap (dotted lines) calculated using Bhattacharyya's affinity for the 50% and 95% utilization distributions of adult and immature gannets, and the null distribution of Bhattacharyya's affinity values estimated by randomly reassigning age class among the 46 individuals 1000 times. (Online version in colour.)

This study provides information on 129 foraging trips for 31 adult gannets and 118 foraging trips for 15 immature gannets, representing data for a total of 393 gannet-days. During this time, adults repeatedly used areas to the northeast and southeast of the breeding colony, while immature birds were much more widely distributed across the North Sea (figure 1; electronic supplementary material, animation S1). Consequently, the overlap in UD between the two groups, estimated using BA (figure 2), was significantly lower than the null expectation for both the 50% and 95% UD contours (BA = 0.23, *p* = 0.04 and BA = 0.69, *p* = 0.01, respectively).

### 3.2. Foraging specialization and proficiency

**Figure 3.**
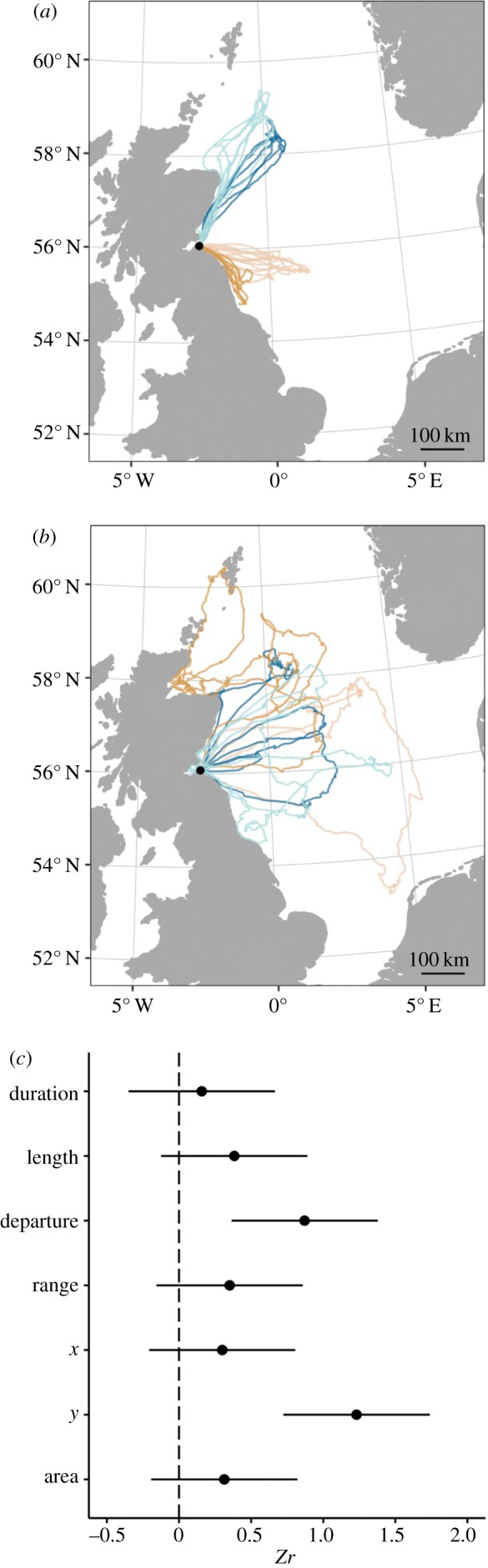
Examples of successive foraging trips by (*a*) four adult and (*b*) four immature gannets, together with (*c*) differences in the point estimates of repeatabilities and 95% confidence intervals for seven measures of foraging trip characteristics (for more information, see Methods). Differences that do not cross the dotted line are significantly different at the *α* = 0.05 level. (Online version in colour.)

Adult gannets were significantly more repeatable than immature birds in the angle at which they departed the colony (*Zr* = 0.87, 95% CI 0.36–1.38) and the *y*-coordinate (latitude) of the terminal point of their foraging trip (*Zr* = 1.23, 95% CI 0.72–1.74), indicating a much higher level of IFSF among adults (figure 3). In addition, foraging trips of adults were much shorter in duration than those of immature birds (median 24 h and 43 h, respectively; table 1; 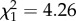, *p* = 0.04) despite there being little difference in the total distance travelled per trip (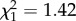, *p* = 0.23), the maximum range from the colony (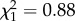, *p* = 0.35) or the area covered at sea per trip (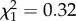, *p* = 0.57; table 1).

**Table 1.** Summary of foraging trip metrics for adult and immature northern gannets *Morus bassanus* tracked from Bass Rock UK.

	adult		immature		
	median	range	median	range	likelihood-ratio test
trip duration (h)	24.4	3.0–56.1	43.0	1.4–411.5	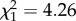 , *p* = 0.04
trip length (km)	629.0	48.8–1201.7	697.4	26.1–4864.8	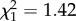 , *p* = 0.23
trip range (km)	239.8	17.6–507.5	283.9	11.0–593.3	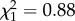 , *p* = 0.35
trip area (km^2^)	7107.4	55.3–34666.9	10545.2	18.9–251190.4	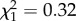 , *p* = 0.57

**Figure 4.**
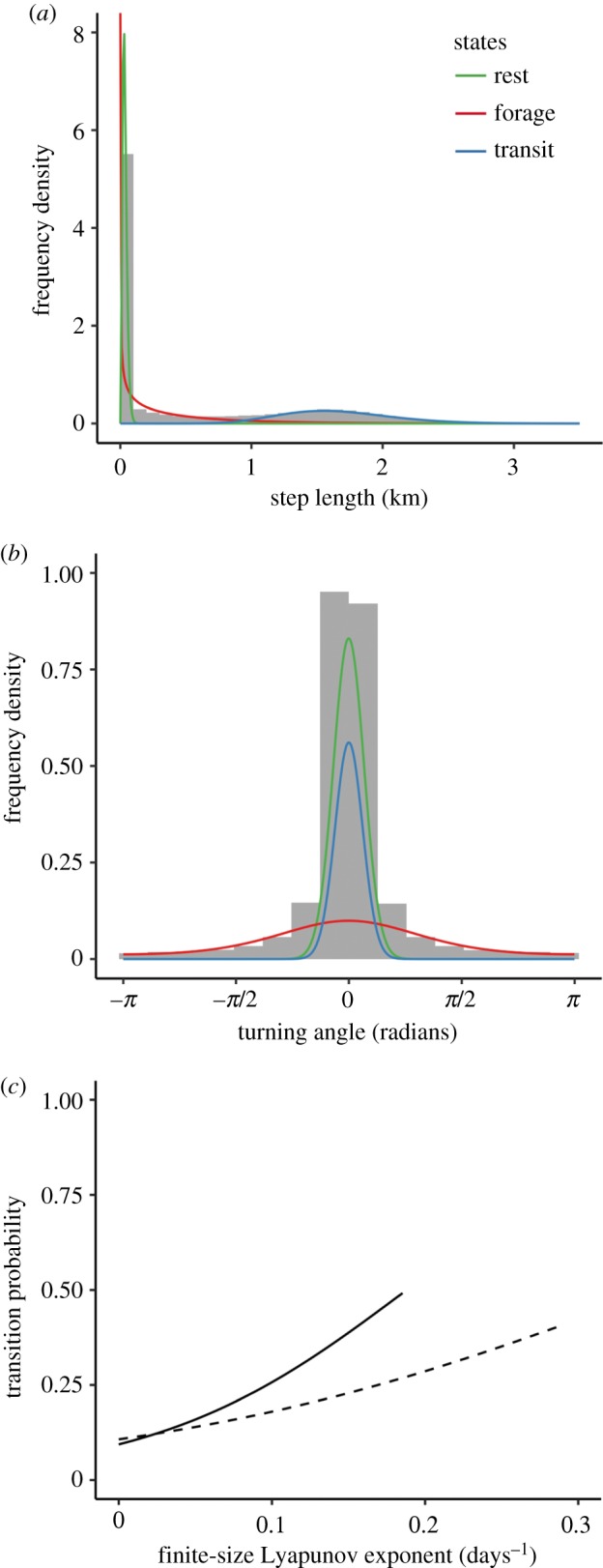
Histograms of (*a*) the observed step length and (*b*) the observed turning angle distributions for GPS-tracked adult and immature gannets. Lines represent the HMM fitted state-dependent distributions, and are coloured according to behavioural mode. (*c*) Model estimated correlation between frontal intensity (finite-size Lyapunov exponent) and the probability of switching from transiting to foraging modes for adult (solid line) and immature (dashed line) gannets. (Online version in colour.)

**Figure 5.**
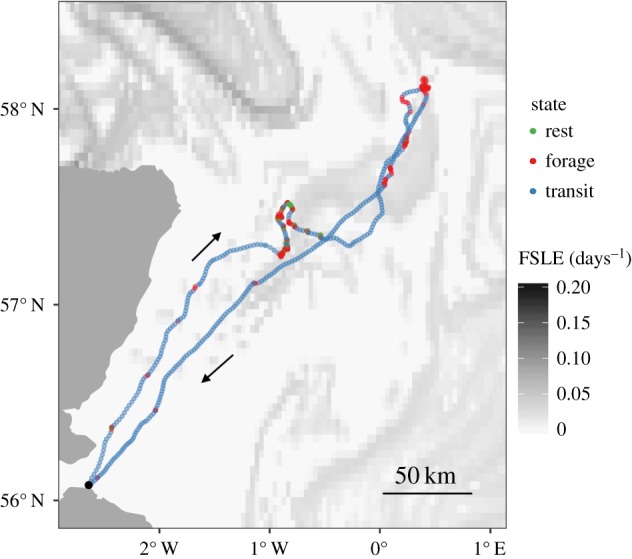
Example overlay of time-matched frontal intensity (finite-size Lyapunov exponent, FSLE) with one adult gannet foraging trip during 19 and 20 June 2015. Darker shading indicates more intense frontal activity, gannet locations are coloured by the Vitterbi-decoded behavioural mode, arrows indicate direction of travel. (Online version in colour.)

The HMM decomposed the tracking data into three distinct states, capturing clearly identifiable movement patterns that we use here as proxies for three behavioural modes: (i) short step lengths and small turning angles (step: 0.03 ± 0.02 km; turn: *μ* = 0, *κ* = 22.3) corresponded with animals resting on the water; (ii) short step lengths and large turning angles (step: 0.41 ± 0.54 km; turn: *μ* = 0, *κ* = 1.0) corresponded with animals foraging and (iii) long step lengths and small turning angles (step: 1.66 ± 0.43 km; turn: *μ* = 0, *κ* = 27.1) corresponded with animals transiting to and from the colony and between foraging sites (figure 4). The AIC of the HMM was greatly improved by including age, FSLE intensity and the interaction between the two (table 2), indicating that adult and immature gannets responded differently to frontal intensity. As predicted, adults exhibited a stronger response to frontal activity than immature birds, and were more likely to switch from transiting to foraging modes as frontal intensity increased (figures 4*c* and 5; electronic supplementary material, animation S2).

**Table 2.** Comparison by AIC of the candidate three-state HMM that included frontal intensity (FSLE) and age as covariates acting on the transition probabilities, and an intercept only model.

	AIC	ΔAIC
FSLE * age	−270679.2	0.0
FSLE + age	−270667.2	12.0
age	−270474.6	204.6
FSLE	−270140.4	538.8
∼1	−269905.0	774.2

During trips, adult and immature gannets spent a similar proportion of the day foraging (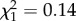, *p* = 0.71; table 3). However, adults spent a smaller proportion of daylight hours resting on the water (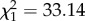, *p* < 0.01) and a greater proportion of time transiting (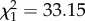, *p* < 0.01) than immature birds. Both adult and immature gannets spent greater than 80% of the night resting on the sea surface.

**Table 3.** Proportion of time spent in each behavioural mode during a foraging trip for adult and immature northern gannets *Morus bassanus* tracked from Bass Rock UK.

	adult		immature		
	median	range	median	range	likelihood-ratio test
foraging	0.363	0.132–0.897	0.318	0.093–0.771	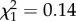 , *p* = 0.71
resting	0.159	0–0.389	0.293	0–0.747	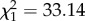 , *p* < 0.01
travelling	0.460	0.063–0.867	0.361	0–0.870	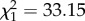 , *p* < 0.01

## 4. Discussion

In this study, our integrated approach revealed novel differences in the foraging specialization and proficiency of adult and immature gannets. In line with our predictions, adults had a much narrower foraging distribution than immature birds and showed greater IFSF. In addition, adults were more likely than immature birds to switch from transiting to foraging modes when encountering areas of high frontal activity. Together these results strongly suggest that the development of IFSF is linked to individuals learning to identify and remember the location of suitable foraging habitat associated with persistent and semi-persistent oceanic fronts.

Adult gannets foraged predominantly to the northeast and southeast of the breeding colony, while immature birds ranged much more widely across the North Sea, with their core foraging distribution (25% and 50% UDs) including extensive areas east of the colony within the central North Sea that were largely ignored by adults (figure 1). A tidal mixing front forms approximately 50 km offshore to the northeast of Bass Rock and has previously been identified as important for gannets foraging from this colony [63]. Both adult and immature gannets visited this region, and also travelled further north to the Fladen Ground, an area that contains a semi-permanent eddy formed from the confluence of the Fair Isle current and East Shetland Atlantic inflow, and also driven in part by local bathymetry [64,65]. Immature gannets then travelled as far north and east as the Norwegian Trench, whereas adults did not. In addition, many more adult than immature birds travelled to the southeast of the breeding colony, using areas of enhanced productivity around the Farn Deeps (figure 1).

Segregation between adult and immature individuals could arise from differences in habitat selection or dietary requirements, mirroring the sexual segregation observed among adults in this population [42,66]. However, while distributions overlapped less than expected by chance, there was nonetheless substantial overlap, particularly northeast of the colony, suggesting that adults and immatures may target similar resources. Immature individuals are less constrained than adults during the breeding season, and so could range further from the breeding colony to target under-used habitat and reduce intraspecific competition [19,38,39] but this suggestion was not supported by the similarity in foraging trip ranges of adults and immature birds in our study (table 1). Hence the narrower foraging distribution of adults most probably arose from more experienced birds choosing a more restricted selection of foraging locations.

Adults had high IFSF and consistently switched from transiting to foraging in response to high frontal density, supporting previous evidence that IFSF among adults results from individuals returning repeatedly to sites characterized by persistent ocean fronts or consistently high fishing activity [28,67]. In contrast to adults, immature birds had both much lower IFSF and a much weaker response to ocean fronts, supporting the hypothesis that IFSF results from individuals learning to identify and relocate such profitable foraging locations [19,35]. Lower IFSF could potentially have been due to immature birds encountering lower intraspecific competition at sea, as a result of their broader foraging distribution [14,68], but this seems unlikely because of their substantial overlap with adults, including short trips in areas of high conspecific density (figure 1), and because longer trips to locations not visited by adults from Bass Rock are likely to have overlapped with birds from adjacent colonies [39]. Hence our data support the notion that IFSF results from learning, with site familiarity being developed in early life during individual exploration or by using social information (for instance, immature gannets frequently follow adults at sea [69]) and subsequently canalized through acquired navigational memory [20,35]. These findings complement recent developments from the physical sciences demonstrating that site fidelity to profitable foraging patches can arise through reinforcement in inhomogeneous environments [70].

Adults had much lower repeatability in trip durations and total distances travelled than in bearings and destinations of trips, as recorded previously [34,71], probably reflecting differences in conditions (e.g. wind) experienced during trips [72] or fine-scale variation in prey availability or individual energy requirements. Overall, adult and immature gannets did not differ in the proportion of time attributed to foraging on each trip. However, given that breeding adults were foraging both for self-maintenance and chick provisioning, while immature birds foraged only to provision themselves, the similarity in the proportion of time spent foraging suggests that in association with greater IFSF and a stronger response to frontal density, adults had greater foraging efficiency than immature birds [73]. This could have resulted from a higher dive rate, a higher success rate or a combination of both. Immature gannets also spent less time in transiting flight and more time resting per trip, which may have been due to lower flight performance [74]. For example, immature Eurasian griffon vultures *Gyps fulvus* have a lower soaring–gliding efficiency, a higher proportion of flapping flight and higher energy expenditure during flight when compared with adults [75].

## 5. Conclusion

Here, we have demonstrated how an integrated approach combining high-resolution bio-logging technology with satellite-derived environmental data in HMMs can provide novel insights into key ecological questions. This approach has been used to identify the principal movement patterns of a marine predator and to reveal age-related differences in how individuals respond when encountering potentially good foraging habitat. Foraging efficiency is well known to increase with age and experience prior to senescence, and the time taken to develop the ability to obtain sufficient food for reproduction, in addition to self-maintenance, may constrain age at first breeding in many long-lived species [10,76]. Our data suggest the development of IFSF through individual learning could play a key role in increasing foraging proficiency, and delayed breeding may be the result of individuals acquiring individual foraging specialization. Further studies, including longitudinal analyses, are now required to quantify the relationship between individual specialization and age at first breeding in long-lived species.

## Supplementary Material

Grecian Supplementary Animation 1

## Supplementary Material

Grecian Supplementary Animation 2

## Data accessibility

The telemetry data are available through the BirdLife International Seabird Tracking Database http://www.seabirdtracking.org.

## References

[1] Charlesworth B 1980 Evolution in age-structured populations. Cambridge, UK: Cambridge University Press.

[2] Stearns SC 1992 The evolution of life histories. Oxford, UK: Oxford University Press.

[3] Lack D 1954 The natural regulation of animal numbers. Oxford, UK: Oxford University Press.

[4] Ashmole NP 1963 The regulation of numbers of tropical oceanic birds. Ibis 103b, 458–473. (10.1111/j.1474-919X.1963.tb06766.x)

[5] Wunderle JM 1991 Age-specific foraging proficiency. Curr. Ornithol. 8, 273–324.

[6] Campioni L, Granadeiro P, Catry P 2016 Niche segregation between immature and adult seabirds: does progressive maturation play a role? Behav. Ecol. 27, 426–433. (10.1093/beheco/arv167)

[7] Buckley FG, Buckley PA 1974 Comparative feeding ecology of wintering abult and juvenile royal terns (*Aves: Laridae, Sterninae*). Ecology 55, 1053–1063. (10.2307/1940355)

[8] Daunt F, Afanasyev V, Adam A, Croxall JP, Wanless S 2007 From cradle to early grave: juvenile mortality in European shags *Phalacrocorax aristotelis* results from inadequate development of foraging proficiency. Biol. Lett. 3, 371–374. (10.1098/rsbl.2007.0157) 17504733 PMC2390668

[9] Orgeret F, Weimerskirch H, Bost C-A 2016 Early diving behaviour in juvenile penguins: improvement or selection processes. Biol. Lett. 12, 20160490 (10.1098/rsbl.2016.0490) 27484650 PMC5014042

[10] Forslund P, Pärt T 1995 Age and reproduction in birds—hypotheses and tests. Trends Ecol. Evol. 10, 374–378. (10.1016/S0169-5347(00)89141-7) 21237076

[11] Tavecchia G, Pradel R, Boy V, Johnson A, Cézilly F 2001 Sex- and age-related variation in survival probability and the cost of the first reproduction in breeding greater flamingos. Ecology 82, 165–174. (10.1890/0012-9658(2001)082%5D0165:SAARVI%5B2.0.CO;2)

[12] Schuppli C, Isler K, Van Schaik CP 2012 How to explain the unusually late age at skill competence among humans. J. Hum. Evol. 63, 843–850. (10.1016/j.jhevol.2012.08.009) 23141772

[13] Araújo MS, Bolnick DI, Layman CA 2011 The ecological causes of individual specialisation. Ecol. Lett. 14, 948–958. (10.1111/j.1461-0248.2011.01662.x) 21790933

[14] Dall SRX, Bell AM, Bolnick DI, Ratnieks FLW 2012 An evolutionary ecology of individual differences. Ecol. Lett. 15, 1189–1198. (10.1111/j.1461-0248.2012.01846.x) 22897772 PMC3962499

[15] Bolnick DI, Svanbäck R, Fordyce JA, Yang LH, Davis JM, Hulsey CD, Forister ML 2003 The ecology of individuals: incidence and implications of individual specialization. Am. Nat. 161, 1–28. (10.1086/343878) 12650459

[16] Estes JA, Riedman ML, Staedler MM, Tinker MT, Lyon BE 2003 Individual variation in prey selection by sea otters: patterns, causes and implications. J. Anim. Ecol. 72, 144–155. (10.1046/j.1365-2656.2003.00690.x)

[17] Lefebvre L 1995 Culturally-transmitted feeding behaviour in primates. Primates 36, 227–239. (10.1007/BF02381348)

[18] Mann J, Sargeant B 2003 Like mother, like calf. In The biology of traditions (eds Fragaszy D, Perry S), pp. 236–266. Cambridge, UK: Cambridge University Press.

[19] Votier SC et al. 2017 Effects of age and reproductive status on individual foraging site fidelity in a long-lived marine predator. Proc. R. Soc. B 284, 20171068 (10.1098/rspb.2017.1068) PMC5543227 28747480

[20] Guilford TC, Freeman R, Boyle D, Dean BJ, Kirk H, Phillips RA, Perrins CM 2011 A dispersive migration in the Atlantic puffin and its implications for migratory navigation. PLoS ONE 6, e21336 (10.1371/journal.pone.0021336) 21799734 PMC3140476

[21] Le Fevre J 1986 Aspects of the biology of frontal systems. Adv. Mar. Biol. 23, 164–299. (10.1016/S0065-2881(08)60109-1)

[22] Yoder JA, Ackleson SG, Barber RT, Flament P, Balch WM 1994 A line in the sea. Nature 371, 689–692. (10.1038/371689a0)

[23] Genin A, Jaffe JS, Reef R, Richter C, Franks PJS 2005 Swimming against the flow: a mechanism of zooplankton aggregation. Science 308, 860–862. (10.1126/science.1107834) 15879218

[24] Cotté C, D'Ovidio F, Chaigneau A, Levy M, Taupier-Letage I, Mate B, Guinet C 2011 Scale-dependent interactions of Mediterranean whales with marine dynamics. Limnol. Oceanogr. 56, 219–232. (10.4319/lo.2011.56.1.0219)

[25] Scales KL, Miller PI, Varo-Cruz N, Hodgson DJ, Hawkes LA, Godley BJ 2015 Oceanic loggerhead turtles *Caretta caretta* associate with thermal fronts: evidence from the canary current large marine ecosystem. Mar. Ecol. Prog. Ser. 519, 195–207. (10.3354/meps11075)

[26] Della Penna A, De Monte S, Kestenare E, Guinet C, D'Ovidio F 2015 Quasi-planktonic behavior of foraging top marine predators. Sci. Rep. 5, 18063 (10.1038/srep18063) 26666350 PMC4678296

[27] Tew Kai ET, Rossi V, Sudre J, Weimerskirch H, Lopez C, Hernandez-Garcia E, Marsac F, Garcon V 2009 Top marine predators track Lagrangian coherent structures. Proc. Natl Acad. Sci. USA 106, 8245–8250. (10.1073/pnas.0811034106) 19416811 PMC2677090

[28] Scales KL, Miller PI, Embling CB, Ingram SN, Pirotta E, Votier SC 2014 Mesoscale fronts as foraging habitats: composite front mapping reveals oceanographic drivers of habitat use for a pelagic seabird. J. R. Soc. Interface 11, 20140679 (10.1098/rsif.2014.0679) 25165595 PMC4191095

[29] Cox SL, Miller PI, Embling CB, Scales KL, Bicknell AWJ, Hosegood PJ, Morgan G, Ingram SN, Votier SC 2016 Seabird diving behaviour reveals the functional significance of shelf-sea fronts as foraging hotspots. R. Soc. open sci. 3, 160317 (10.1098/rsos.160317) 27703698 PMC5043317

[30] Miller PI, Scales KL, Ingram SN, Southall EJ, Sims DW 2015 Basking sharks and oceanographic fronts: quantifying associations in the north-east Atlantic. Funct. Ecol. 29, 1099–1109. (10.1111/1365-2435.12423)

[31] De Monte S, Cotté C, d'Ovidio F, Lévy M, Le Corre M, Weimerskirch H 2012 Frigatebird behaviour at the ocean–atmosphere interface: integrating animal behaviour with multi-satellite data. J. R. Soc. Interface 9, 3351–3358. (10.1098/rsif.2012.0509) 22951344 PMC3481590

[32] Belkin IM, Cornillon PC, Sherman K 2009 Fronts in large marine ecosystems. Prog. Oceanogr. 81, 223–236. (10.1016/j.pocean.2009.04.015)

[33] Nelson BN 2002 The atlantic gannet, 2nd edn. Great Yarmouth, UK: Fenix Books.

[34] Patrick SC et al. 2014 Individual differences in searching behaviour and spatial foraging consistency in a central place marine predator. Oikos 123, 33–40. (10.1111/j.1600-0706.2013.00406.x)

[35] Wakefield ED, Cleasby IR, Bearhop S, Bodey TW, Davies RD, Miller PI, Newton J, Votier SC, Hamer KC 2015 Long-term individual foraging site fidelity—why some gannets don't change their spots. Ecology 96, 3058–3074. (10.1890/14-1300.1) 27070024

[36] Fagan W et al. 2013 Spatial memory and animal movement. Ecol. Lett. 16, 1316–1329. (10.1111/ele.12165) 23953128

[37] Patterson TA, Basson M, Bravington MV, Gunn JS 2009 Classifying movement behaviour in relation to environmental conditions using hidden Markov models. J. Anim. Ecol. 78, 1113–1123. (10.1111/j.1365-2656.2009.01583.x) 19563470

[38] Votier SC, Grecian WJ, Patrick SC, Newton J 2011 Inter-colony movements, at-sea behaviour and foraging in an immature seabird: results from GPS-PPT tracking, radio-tracking and stable isotope analysis. Mar. Biol. 158, 355–362. (10.1007/s00227-010-1563-9)

[39] Wakefield ED et al. 2013 Space partitioning without territoriality in gannets. Science 341, 68–70. (10.1126/science.1236077) 23744776

[40] Phillips RA, Xavier JC, Croxall JP 2003 Effects of satellite transmitters on albatrosses and petrels. Auk 120, 1082–1090. (10.1642/0004-8038(2003)120%5B1082:EOSTOA%5D2.0.CO;2)

[41] Hamer KC, Humphreys EM, Garthe S, Hennicke J, Peters G, Grémillet D, Phillips RA, Harris MP, Wanless S 2007 Annual variation in diets, feeding locations and foraging behaviour of gannets in the North Sea: flexibility, consistency and constraint. Mar. Ecol. Prog. Ser. 338, 295–305. (10.3354/meps338295)

[42] Cleasby IR, Wakefield ED, Bodey TW, Davies R, Patrick SC, Newton J, Votier SC, Bearhop S, Hamer KC 2015 Sexual segregation in a wide-ranging marine predator is a consequence of habitat selection. Mar. Ecol. Prog. Ser. 518, 1–12. (10.3354/meps11112)

[43] Boffetta G, Lacorata G, Redaelli G, Vulpiani A 2001 Detecting barriers to transport: a review of different techniques. Phys. D Nonlinear Phenom. 159, 58–70. (10.1016/S0167-2789(01)00330-X)

[44] d'Ovidio F, Isern-Fontanet J, López C, Hernández-García E, García-Ladona E 2009 Comparison between Eulerian diagnostics and finite-size Lyapunov exponents computed from altimetry in the Algerian basin. Deep. Res. I Oceanogr. Res. Pap. 56, 15–31. (10.1016/j.dsr.2008.07.014)

[45] Nel D, Lutjeharms JRE, Pakhomov EA, Ansorge IJ, Ryan PG, Klages NTW 2001 Exploitation of mesoscale oceanographic features by grey-headed albatross *Thalassarche chrysostoma* in the southern Indian Ocean. Mar. Ecol. Ser. 217, 15–26. (10.3354/meps217015)

[46] Hyrenbach KD, Veit RR, Weimerskirch H, Hunt GL 2006 Seabird associations with mesoscale eddies: the subtropical Indian Ocean. Mar. Ecol. Ser. 324, 271–279. (10.3354/meps324271)

[47] Carter MID et al. 2016 GPS tracking reveals rafting behaviour of Northern Gannets (*Morus bassanus*): implications for foraging ecology and conservation. Bird Study 3657, 1–13. (10.1080/00063657.2015.1134441)

[48] Calenge C 2006 The package adehabitat for the R software: a tool for the analysis of space and habitat use by animals. Ecol. Modell. 197, 516–519. (10.1016/j.ecolmodel.2006.03.017)

[49] Bhattacharyya A 1943 On a measure of divergence between two statistical populations defined by their probability distributions. Bull. Calcutta Math. Soc. 35, 99–109.

[50] Nakagawa S, Schielzeth H 2010 Repeatability for Gaussian and non-Gaussian data: a practical guide for biologists. Biol. Rev. 85, 935–956. (10.1111/j.1469-185X.2010.00141.x) 20569253

[51] English S, Nakagawa S, Clutton-Brock TH 2010 Consistent individual differences in cooperative behaviour in meerkats (*Suricata suricatta*). J. Evol. Biol. 23, 1597–1604. (10.1111/j.1420-9101.2010.02025.x) 20492087

[52] Agostinelli C, Lund U 2013 R package ‘circular’: circular statistics. See https://r-forge.r-project.org/projects/circular/.

[53] Lessells CM, Boag PT 1987 Unrepeatable repeatabilities: a common mistake. Auk 104, 116–121. (10.2307/4087240)

[54] Becker WA 1992 Manual of quantitative genetics, 4th edn. Pullman, WA: Academic Enterprises.

[55] Michelot T, Langrock R, Patterson TA 2016 moveHMM: an R package for the statistical modelling of animal movement data using hidden Markov models. Methods Ecol. Evol. 7, 1308–1315. (10.1111/2041-210X.12578)

[56] Pohle J, Langrock R, van Beest FM, Schmidt NM 2017 Selecting the number of states in hidden markov models: pragmatic solutions illustrated using animal movement. J. Agric. Biol. Environ. Stat. 22, 270–293. (10.1007/s13253-017-0283-8)

[57] Bennison A, Bearhop S, Bodey TW, Votier SC, Grecian WJ, Wakefield ED, Hamer KC, Jessopp M 2017 Search and foraging behaviors from movement data: a comparison of methods. Ecol. Evol. 8, 13–24. (10.1002/ece3.3593) 29321847 PMC5756868

[58] Zucchini W, MacDonald IL 2009 Hidden Markov models for time series: an introduction using R. London, UK: Chapman and Hall.

[59] Towner AV, Leos-Barajas V, Langrock R, Schick RS, Smale MJ, Kaschke T, Jewell OJD, Papastamatiou YP 2016 Sex-specific and individual preferences for hunting strategies in white sharks. Funct. Ecol. 30, 1397–1407. (10.1111/1365-2435.12613)

[60] Leos-Barajas V, Photopoulou T, Langrock R, Patterson TA, Watanabe YY, Murgatroyd M, Papastamatiou YP 2017 Analysis of animal accelerometer data using hidden Markov models. Methods Ecol. Evol. 8, 161–173. (10.1111/2041-210X.12657)

[61] Bates D, Maechler M, Bolker BM, Walker S 2015 lme4: Linear mixed-effects models using Eigen and S4.

[62] R Core Team. 2016 R: a language and environment for statistical computing. Vienna, Austria: R Foundation for Statistical Computing.

[63] Hamer KC, Humphreys EM, Magalhães MC, Garthe S, Hennicke J, Peters G, Grémillet D, Skov H, Wanless S 2009 Fine-scale foraging behaviour of a medium-ranging marine predator. J. Anim. Ecol. 78, 880–889. (10.1111/j.1365-2656.2009.01549.x) 19426254

[64] Svendsen E, Sætre R, Mork M 1991 Features of the northern North Sea circulation. Cont. Shelf Res. 11, 493–508. (10.1016/0278-4343(91)90055-B)

[65] Turrell WR 1992 New hypotheses concerning the circulation of the northern North Sea and its relation to North Sea fish stock recruitment. ICES J. Mar. Sci. 49, 107–123. (10.1093/icesjms/49.1.107)

[66] Stauss C et al. 2012 Sex-specific foraging behaviour in northern gannets *Morus bassanus*: incidence and implications. Mar. Ecol. Prog. Ser. 457, 151–162. (10.3354/meps09734)

[67] Patrick SC, Bearhop S, Bodey TW, Grecian WJ, Hamer KC, Lee J, Votier SC 2015 Individual seabirds show consistent foraging strategies in response to predictable fisheries discards. J. Avian Biol. 46, 431–440. (10.1111/jav.00660)

[68] Svanbäck R, Bolnick DI 2007 Intraspecific competition drives increased resource use diversity within a natural population. Proc. R. Soc. B 274, 839–844. (10.1098/rspb.2006.0198) PMC2093969 17251094

[69] Votier SC, Bicknell AWJ, Cox SL, Scales KL, Patrick SC 2013 A bird's eye view of discard reforms: bird-borne cameras reveal seabird/fishery interactions. PLoS ONE 8, e57376 (10.1371/journal.pone.0057376) 23483906 PMC3590202

[70] Falcón-Cortés A, Boyer D, Giuggioli L, Majumdar SN 2017 Localization transition induced by learning in random searches. Phys. Rev. Lett. 119, 140603 (10.1103/PhysRevLett.119.140603) 29053283

[71] Hamer KC, Phillips RA, Hill JK, Wanless S, Wood AG 2001 Contrasting foraging strategies of gannets *Morus bassanus* at two North Atlantic colonies: foraging trip duration and foraging area fidelity. Mar. Ecol. Ser. 224, 283–290. (10.3354/meps224283)

[72] Amélineau F, Péron C, Lescroël A, Authier M, Provost P, Grémillet D 2014 Windscape and tortuosity shape the flight costs of northern gannets. J. Exp. Biol. 217, 876–885. (10.1242/jeb.097915) 24622894

[73] Fayet A, Freeman R, Shoji A, Padget O, Perrins CM, Guilford TC 2015 Lower foraging efficiency in immatures drives spatial segregation with breeding adults in a long-lived pelagic seabird. Anim. Behav. 110, 79–89. (10.1016/j.anbehav.2015.09.008)

[74] Riotte-Lambert L, Weimerskirch H 2013 Do naive juvenile seabirds forage differently from adults? Proc. R. Soc. B 280, 20131434 (10.1098/rspb.2013.1434) PMC3757974 23926153

[75] Harel R, Horvitz N, Nathan R 2016 Adult vultures outperform juveniles in challenging thermal soaring conditions. Sci. Rep. 6, 27865 (10.1038/srep27865) 27291590 PMC4904409

[76] Krüger O 2005 Age at first breeding and fitness in goshawk *Accipiter gentilis*. J. Anim. Ecol. 74, 266–273. (10.1111/j.1365-2656.2004.00920.x)

